# Functional Characterization of a Hexose Transporter from Root Endophyte *Piriformospora indica*

**DOI:** 10.3389/fmicb.2016.01083

**Published:** 2016-07-22

**Authors:** Mamta Rani, Sumit Raj, Vikram Dayaman, Manoj Kumar, Meenakshi Dua, Atul K. Johri

**Affiliations:** ^1^School of Life Sciences, Jawaharlal Nehru UniversityNew Delhi, India; ^2^School of Environmental Sciences, Jawaharlal Nehru UniversityNew Delhi, India

**Keywords:** root-endophyte, *Piriformospora indica*, MFS superfamily, hexose transporter, glucose uptake

## Abstract

Understanding the mechanism of photosynthate transfer at symbiotic interface by fungal monosaccharide transporter is of substantial importance. The carbohydrate uptake at the apoplast by the fungus is facilitated by PiHXT5 hexose transporter in root endophytic fungus *Piriformospora indica.* The putative PiHXT5 belongs to MFS superfamily with 12 predicted transmembrane helices. It possess sugar transporter PFAM motif (PF0083) and MFS superfamily domain (PS50850). It contains the signature tags related to glucose transporter GLUT1 of human erythrocyte. *PiHXT5* is regulated in response to mutualism as well as glucose concentration. We have functionally characterized *PiHXT5* by complementation of *hxt-null* mutant of *Saccharomyces cerevisiae* EBY.VW4000. It is involved in transport of multiple sugars ranging from D-glucose, D-fructose, D-xylose, D-mannose, D-galactose with decreasing affinity. The uncoupling experiments indicate that it functions as H^+^/glucose co-transporter. Further, pH dependence analysis suggests that it functions maximum between pH 5 and 6. The expression of *PiHXT5* is dependent on glucose concentration and was found to be expressed at low glucose levels (1 mM) which indicate its role as a high affinity glucose transporter. Our study on this sugar transporter will help in better understanding of carbon metabolism and flow in this agro-friendly fungus.

## Introduction

Mycorrrhiza represents 400–100 million year of plant–fungus co-evolution due to nutritional dependency on each other. It has been credited with the most predominant association (70–90% of terrestrial plant species) in nature (Smith and Read, 2010). In comparison to the direct uptake of phosphate (Pi) by plant root, mycorrhiza dependent pathway is proved to be most effective pathway to scavenge soil Pi and to deliver it to the root cortical cells. Most of the mycorrhizal fungi, belongs to the phylum Glomeromycota and Basidiomycota, are soil inhabitants and utilize up to 30% of photosynthates in exchange of nutrient supplied from soil. *Piriformospora indica* is an root endophytic fungus that belongs to order sabacinale (Varma et al., 1999; Weiss et al., 2011). *P. indica* has several beneficiary effects on their host plants which include nutrient transport like phosphate, increase in biomass and grain yield and provide resistance to host plant against various abiotic and biotic stresses (Waller et al., 2005; Baltruschat et al., 2008; Sherameti et al., 2008; Kumar et al., 2009; Oelmüller et al., 2009; Vadassery et al., 2009; Sun et al., 2010; Yadav et al., 2010; Jogawat et al., 2013; Trivedi et al., 2013). As the arbuscular mycorrhizal fungi (AMF) cannot be cultured axenically, therefore their use in sustainable agriculture for crop improvement was not so successful. *P. indica* has an advantage over AMF as it has a broad-host spectrum as compared to AMF (Peskan-Berghofer et al., 2004) and can be propagated axenically. Its stable transformation system has been developed, so it can be easily manipulated genetically to understand different biological mechanisms and can be used as a model to study plant–fungal interaction (Yadav et al., 2010). *P. indica* grows progressively inter- and intra-cellularly and forms coiled structure similar to arbuscules of AMF (Gianinazzi-Pearson, 1996; Schäfer and Kogel, 2009). It creates a microsymbiont that involves the infolding of the periarbuscular membrane of the host plant around the fungal wall, by forming an apoplastic space enclosed by symbiotic interface (Parniske, 2004; Harrison, 2005; Felle et al., 2009). Here, the bidirectional nutrient exchange involving phosphate and carbohydrate takes place (Shachar-Hill et al., 1995; Karandashov and Bucher, 2005). Symbiotic relation is so important that mycorrhizal plants can solely depend on phosphate acquisition by this route (Liu et al., 1998; Smith et al., 2003). Carbohydrate is the price paid by plant in exchange of phosphate supplied (Bonfante and Anca, 2009; Plett and Martin, 2011). It has been proposed that at root-fungus symbiotic interface, first the passive diffusion of carbohydrates and Pi occurs via plant and fungal plasma membrane respectively, followed by uptake of these nutrients by active transport driven by H^+^-ATPase(s) (Smith et al., 2001). However, the mechanism related to the carbohydrate and Pi transportation across symbiotic interface is still unknown. In case of mycorrhizal fungi, very less information is available on carbohydrate transporters active during symbiosis. A high-affinity glucose transporter *AmMST1* was characterized from ectomycorrhizal fungi *Amanita muscaria* (Smith et al., 2001), another high-affinity hexose transporter *TbHXT1* was characterized from *Tuber borchii Vittadini* (Polidori et al., 2007). In *Laccaria bicolor* genome, 15 putative hexose transporter genes are identified. Three of the above genes (*LbMST1.2, LbMST1.3*, and *LbMST3.1*) have been characterized as high-affinity glucose transporters (Lopez-Pedrosa et al., 2006). Only two reports are available related to the hexose transporters from AMF which are involved in the symbiosis like *GpMST1* from symbiotic fungus *Geosiphon pyriformis* and *MST2* from *Glomus intraradices* (Schubler et al., 2006; Helber et al., 2011), both of these proteins are high affinity glucose transporters with a broad substrate specificity for different monosaccharides.

Recent genome sequencing of *P. indica* genome has revealed presence of 19 putative hexose transporter genes (Zuccaro et al., 2011). The presence of high number of putative hexose transporter genes suggest that there are different type of hexose uptake system to function under different conditions. Till date, no report is available on hexose transporters in mutualistic endophyte *P. indica.* The characterization of hexose transporter genes are important in order to understand the mechanism of carbohydrate uptake during symbiosis. For the first time, we have characterized a monosaccharide transporter *PiHXT5* (accession number *CCA71201.1)* from *P. indica*. *PiHXT5* was found upregulated during colonization of *P. indica* with maize plant as compare to axenically grown fungus. Functional studies suggest that *PiHXT5* encode for a high-affinity hexose transporter and its activity depends on proton gradient and pH.

## Materials and Methods

### Fungal, Plant Materials, and Growth Conditions

*Piriformospora indica* (Verma et al., 1998) was used throughout the study. *Zea mays* (var. pro33) was used for plant–fungus interaction (Yadav et al., 2010). *Escherichia coli* DH5-α was used for cloning purposes (Inoue et al., 1990). *Saccharomyces cerevisiae* hexose uptake mutant EBYVW.4000 (*MATa* _*hxt1-17* _*gal2* _*stl1* _*agt1* _*mph2*_*mph3 leu2-3, 112 ura3-52 trp1-289 his3-*_*1 MAL2-8c SUC2*; Wieczorke et al., 1999) was used for functional characterization and kinetic studies of *PiHXT5*. *P. indica* culture was maintained in modified *Aspergillus* minimal medium (Hill and Kafer, 2001) or in modified MN medium (Bécard and Fortin, 1988). The yeast strain EBY.VW4000 unless otherwise mentioned, was maintained on YPD medium supplemented with 2% maltose instead of glucose. Plasmid p112A1NE transformed *E. coli* DH5-α was maintained on LB agar plates containing ampicillin (Sigma). *Z. mays* seeds were washed with Tween-20 detergent and later surface sterilized with 75% ethanol for 2 min followed by treatment with 0.75% NaClO for 5 min and after washing six times with sterile distilled water, seeds were treated at 60°C in sterile water for 5 min (Kumar et al., 2009). Seeds were kept on water agar plates for germination (0.8% Bacto Agar; Difco, Detroit, MI, USA) at 25°C in the dark (Gamborg and Phillips, 2013).

### Bioinformatics Analysis

For identification of hexose transporters in *P. indica* genome, blastX algorithm^1^ was used. For modeling *PiHXT5* transmembrane (TM) domains, TM helix segments were predicted with various computer programs; e.g., SOSUI, HMMTOP, TMHMM, and TMpred (Von Heijne, 1994; Hirokawa et al., 1998; Krogh et al., 2001; Tusnady and Simon, 2001). A consensus of 12 TM helices emerged and the boundary of each predicted helix was identified based on empirical rules derived from known membrane protein structures. Sequence alignments were done with ClustalW and BLOSUM62 with a gap penalty of 10 for insertion and 5 for extension (Henikoff and Henikoff, 1992; Thompson et al., 1994). Multiple Sequence Alignment were performed to search the conserved signature tags of the glucose transporters derived from *Homo sapiens (GLUT1, GLUT2, GLUT3)* and *E. coli (XylE;* Iancu et al., 2013; Deng et al., 2014; Wisedchaisri et al., 2014).

### Expression Analysis of *PiHXT5, PiHXT8*, and *PiHXT9* during Colonization

Expression of *P. indica PiHXT5* (GenBank ID: CAFZ01000110.1*), PiHXT8* (GenBank ID: CAFZ01000044.1), and *PiHXT9* (GenBank ID: CAFZ01000021.1) during colonization with maize plants were compared with axenically grown *P. indica*. For this purpose, *P. indica* was grown in *Aspergillus* minimal media in 111 mM (or 2% w/v) glucose and 10 mM phosphate (Hill and Kafer, 2001) for 7 days and then subsequently transferred to MN media (Bécard and Fortin, 1988) with 111 mM glucose and low phosphate (10 μM KH_2_PO_4_) and grown for 15 days. For colonization, radicals germinated seedlings were first submerged into macerated *P. indica* and incubated for 4 days on water agar and finally transferred to MN with low phosphate (10 μM) and allowed to grow for 15 days, whereas in case of control plants autoclaved double distilled water was used. The MN medium was changed weekly. At 15 days of colonization, maize roots were harvested, and RNA was isolated from these roots. The plants were maintained in an environmentally controlled green house at 30 ± 2°C and 16 h light/8 h dark and relative humidity 60–70%, with light intensity of 1000 Lux. To check colonization, root segments (1.0 cm approximately) were heated on 10% KOH for 15 min, treated with 1 N HCl and were stained with 0.05% trypan blue overnight at RT(or stained at 60°C for 1 h) and mounted on lactophenol (Kumar et al., 2009). Observation was done under light microscope (Leica Microscope. Type 020-518.500, Germany). Percent colonization was calculated for the inoculated plants using the following formula (Mcgonigle et al., 1990).

(1) $$\mathrm{Percent}\;\mathrm{colonization}=\frac{\mathrm{No}.\;\mathrm{of}\;\mathrm{colonized}\;\mathrm{root}\;\mathrm{segments}}{\mathrm{Total}\;\mathrm{number}\;\mathrm{of}\;\mathrm{segments}}\;\times 100$$

Further, to study the effect of different glucose concentration on expression pattern of *PiHXT5, P. indica* was grown in KF medium at 10 mM Phosphate and 111 mM glucose for 7 days and subsequently transferred to MN media (Bécard and Fortin, 1988) containing 10 mM phosphate and different glucose concentrations ranging from very low to high glucose concentration (1 and 500 mM) and grown for 5 days at 110 rpm and 30 ± 2°C temperature. After 5 days of growth, *P. indica* culture was harvested and RNA was isolated. For quantitative real time PCR (qRT-PCR) analysis the expression of *PiHXT5* in all these conditions was compared with *P. indica* grown in optimum glucose concentration (111 mM glucose).

### Isolation of RNA, cDNA Synthesis, and qRT-PCR

Total RNA was isolated from *P. indica* colonized maize plant roots and from axenically grown fungus. Samples were harvested and washed with sterile water and frozen in liquid nitrogen. These tissue samples were then crushed in liquid nitrogen and RNA was isolated using TRIzol reagent according to the protocol provided by the manufacturer (Invitrogen, USA). The concentration of RNA was quantified by Nanodrop-2000 (Thermoscientific) spectrophotometer and RNA integrity was checked on denaturing formaldehyde agarose gel with 1.2% agarose by fractionating the 10 μg of total RNA. For cDNA synthesis 1.5 μg of total RNA was treated with DNase1 (Thermoscientific) and cDNA was synthesized using Thermoscientific First strand synthesis Kit. Real time PCR was performed with gene specific primer pairs (**Table 1**) and SYBR Green I (TakaRa) using ABI 7500 Real-Time PCR System (Applied Biosystems) according to manufacturer’s instructions. The cycling conditions were as follows: denaturation was done at 94°C for 5 min for one cycle. Subsequently, for 40 cycles denaturation was done at 95°C for 15 s, annealing was done at 57°C for 30 s and extension was done at 68°C for 30 s. *P. indica* translational elongation factor gene *(PiTef)* was used as a reference.

**Table 1:** Oligonucleotides used in this study.

Primer	Sequence	Purpose
*PiHXT5*	For 5′-ATGGCCAGCTTCTTCAACCAA-3′ Rev 5′-CGCCAGCTCTTCCAGTGTTCT-3′	qRT-PCR
*PiHXT8*	For 5′-ATGCCTGGTGGTGGTGCAGT-3′ Rev 5′-TTAGACCTTCTCTTGTGCACT-3′	qRT-PCR
*PiHXT9*	For 5′-TCTCCGGTGTCAAGGAAATGA-3′ Rev 5′-AGAAGATGAGGCAAGCACCGA-3′	qRT-PCR
*PiTEF*	For 5′-TCGTCGCTGTCAACAAGATG-3′ Rev 5′–GAGGGCTCGAGCATGTTGT-3′	qRT-PCR
*PiHXT5*	For 5′-AACTGCAGATGGGTGGCGACATTGC-3′ Rev 5′-CGGGATCCTTACGCCTTGTCCTCTGCT-3′	Cloning of *PiHXT5*
*T7*	For 5′-TAATACGACTCACTATAGGG-3′	Sequencing of *PiHXT5*
*SP6*	Rev 5′-ATTTAGGTGACACTATAG-3′	Sequencing of *PiHXT5*
*PiHXT5*	For 5′-GCACCTTTGCGATGCCAAA-3′ Rev 5′-CTTGCTTATCCTTCGCAATTGT-3′	Sequencing of *PiHXT5*
AP1	5′-CTAATACGACTCACTATAGGGCAAGCAGTGGTATCAACGCAGAGT-3′ 5′-CTAATACGACTCACTATAGGGC-3′	5′ and 3′ RACE analysis
AP2	5′-AAGCAGTGGTATCAACGCAGAGT-3′	5′ and 3′ RACE analysis
GSP1	5′-AGCTCCCGCAGACAATGGA-3′	5′ RACE analysis
GSP1′	5′-ATGGCCAGCTTCTTCAACCAA-3′	3′ RACE analysis (Primary PCR)
GSP2′	5′-AGGTGAGAAAGAGGCAGGCAA-3′	3′ RACE analysis (Nested PCR)

### Isolation of *PiHXT5* Coding DNA Sequence (CDS)

*PiHXT5* CDS was amplified with gene specific primer pair (**Table 1**) using *Pfu* polymerase (Thermoscientific). The cycling conditions were as follows: 94°C for 3 min (one cycle), 94°C for 40 s, 59°C for 30 s, 72°C for 3 min (35 cycles) and 72°C for 5 min (one cycle). Amplified PCR product was cloned into pGEM-T Easy vector (Promega). The pGEMT-*PiHXT5* construct was transformed in *E. coli* DH5-α cell and transformants were confirmed by colony PCR with gene specific primers (**Table 1**). Plasmid was isolated from the positive clones and confirmed by restriction digestion using *Pst*1 and *Bam*H1. Further cloning was confirmed by sequencing.

### Rapid Amplification of cDNA Ends (RACE)

The full-length cDNA sequences of *PiHXT5* were obtained after rapid amplification of cDNA ends (RACE) using the protocols of the SMART (Clontech) kits. The 5′ RACE library was prepared by the incorporation of a “smart oligo” at the 5′-end of the reverse-transcribed cDNA. 5′-end of the *PiHXT5* gene was amplified from 5′ RACE library using a gene-specific oligonucleotide (GSP1). Further amplicon specificity is enhanced in secondary PCR using the same gene-specific oligonucleotide (GSP1). The GSP1 primer is located at 312–331 bp downstream of the ATG codon. For 3′ RACE library, the 3′-end of the *PiHXT5* was amplified from 3′ RACE library using gene specific oligonucleotide (GSP1′ and GSP2′). The GSP1′ and GSP2′ primers are located at 1288–1308 bp and 1463–1483 downstream of the ATG codon. Primers were designed such that of 331 bp along with 5′ UTR were amplified. Similarly, amplicon of 312 and 137 bp with 3′ UTR in primary and nested PCR, were obtained respectively. Amplicons were analyzed by 1.5% agarose gel electrophoresis and purified using the GeneJet gel extraction kit (Thermo scientific, Lithuania, EU). Purified 5′ RACE and 3′ RACE fragment was cloned into the T-A cloning vector pGEM-T Easy and 10 random colonies of each were sequenced.

### CLUSTALW2 and Phylogenetic Analysis

Phylogenetic relationship study was done including deduced amino acid sequence of *PiHXT5* from *P. indica* and the sugar transporters sequences from other fungi, plants and human. Sequences were obtained from GenBank databases. Multiple sequence alignment was done using CLUSTALW2^2^ and MULTIALIN algorithum (Corpet, 1988). Phylogenetic studies were done using neighbor-joining method by MEGA 5 software. Bootstrap step was conducted using 1000 replicates to support the inferred clades (Felsenstein, 1985). The tree is drawn to scale having branch lengths in the same units as those of the evolutionary distances used to deduce the phylogenetic tree. Poisson correction method (Zuckerkandl and Pauling, 1965) was used to compute evolutionary distances which represent units of the number of amino acid substitutions per site. The rate variation among sites was modeled with a gamma distribution (shape parameter = 1). We have used 54 amino acid sequences for the analysis purpose and a total of 296 positions were used for phylogenetic analysis. The sequence of the sugar transporters ST1 from *Medicago truncatula* and *Z. mays* were used as outgroups.

### Complementation and Growth Assay

For complementation, *S. cerevisiae* hexose transporter deficient strain EBY.VW4000 was used. The pGEM-T-*PiHXT5* construct digested with *Pst*1 and *Bam*H1 and the *PiHXT5* fragment obtained was sub-cloned into p112A1NE vector (Leggewie et al., 1997) which was previously treated with same enzymes. The ligation was done using T4 DNA ligase (Thermoscientific). *E. coli* DH5-α cells were transformed with p112A1NE-*PiHXT5* construct and recombinant clones were confirmed by colony PCR with *PiHXT5* specific primers. Further cloning was confirmed by digestion of plasmid with *Pst*1 and *Bam*H1 and confirmed by sequencing. EBY.VW4000 mutant was transformed with p112A1NE-*PiHXT5* construct by LiCl-PEG method (Gietz and Schiestl, 1991; Riesmeier et al., 1992). Procedures and medium used for growth and selection of transformants were similar as described (Bun-Ya et al., 1991). The transformants were first selected on their ability to grow on YNB medium deficient in tryptophan (Boles and Hollenberg, 1997) containing 1% maltose as sole carbon source and later the single colonies of these transformants were transferred to the same medium with 2% glucose. The growth of *PiHXT5* containing yeast was compared with EBY.VW4000 containing only p112A1NE vector. The recombinant clones which were able to grow on glucose were selected for further experiments.

### ^14^C-Glucose Transport Assay and Biochemical Kinetics Characterization

For kinetic study, yeast cells were grown to an OD_600_ of 0.5–0.7 in YNB medium without tryptophan and with 2% maltose. The cells were harvested by centrifugation at 4000 rpm for 5 min. Further, the harvested cells were washed two times with buffer A (50 mM potassium phosphate, 0.6 M sorbitol, pH 6.5) and re-suspended in the same buffer to a final concentration of 10^9^ cell/ml. ^14^C-glucose uptake was done as described previously (Polidori et al., 2007; Wahl et al., 2010). To start sugar uptake 100 μl of yeast cell suspension was mixed with 100 μl of D-glucose (5, 25, 100, 500, 1, and 10 mM non-radioactive D-glucose plus 5 μM ^14^C-glucose, specific activity 0.2 μCi/μL). After 30, 60, 120, and 240 s, 50 μl aliquots from each reaction mix were transferred to 4 ml of ice-cold buffer A, to block sugar uptake. Cells were filtered onto a GF/C grade filter paper (Whatman Glass microfiber filters), and washed twice with 10 ml of ice-cold buffer. Then the filters were transferred into scintillation vials containing 5 ml of scintillation cocktail ‘O’ (CDH) and the radioactivity was measured with scintillation counter (Liquid Scintillation Analyzer TRI-CARB 2100TR; Packard). Amount of the glucose retained on the filters was determined by using the specific activity of the substrate (^14^C-glucose) solutions which are used during the experiment. The competition experiments were performed in the presence of different unlabeled substrates D-glucose, D-fructose, D-mannose, D-galactose, D-xylose. In the competition experiments, yeast strain EBYVW.4000 expressing *PiHXT5* was incubated (1 min at 30°C) with 25 μM ^14^C-glucose in the presence of five-fold excess of different sugars. The uptake rate of ^14^C-glucose is reported in percentage of control (without competitor), and 100% uptake corresponds to 225 pmol.min^-1^.10^8^ cells^-1^. All uptake experiments were repeated with independent samples at least three times. Kinetic studies of *PiHXT5* were measured by uptake rate as a function of glucose concentration. The kinetic parameters were determined using the Michaelis–Menten kinetics plot, and all the data were analyzed by non-linear regression using the Enz-Fitter program. The standard errors were evaluated by the same program. Sugar uptake inhibition experiments were performed using the uncoupling compound 2,4-dinitrophenol (DNP) and H^+^-ATPase inhibitor sodium vanadate at a final concentration of 100 μM, respectively, in the presence of 25 μM ^14^C-glucose. pH dependence of *PiHXT5* for glucose transportation was analyzed by adjusting the potassium phosphate buffer at different pH values ranging from 2 to 8 and then ^14^C-glucose uptake was measured as described above, at pH 2–8.

## Results

### Identification of Sugar Transporters from *P. indica* Genome

**FIGURE 1:**
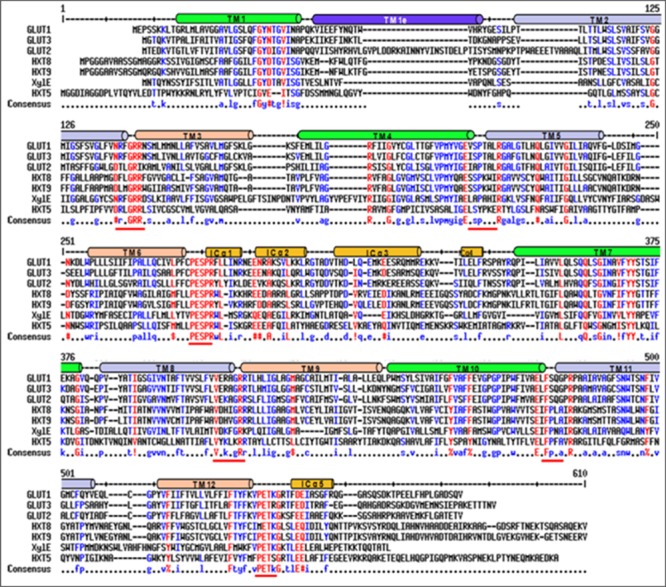
**Sequence alignment of *Piriformospora indica* PiHXT5, PiHXT8, and PiHXT9 with hGLUT1-3 and XylE.** Secondary structural elements of GLUT1 are indicated above the sequence alignment. Invariant and highly conserved amino acids are red and blue, respectively. The conserved sugar porter family signature motifs are underscored with red lines.

Blastx search of first *P. indica* (Genus ID: 65672) genome sequencing project^3^ using already characterized fungal hexose transporters such as *GiMST2* from *G. intraradices, GpMST1* from *G. pyriformis, ScHXT1* from *S. cerevisiae* and *TbHXT1* from *Tuber borchii* as query, a total of 19 putative sugar transporter sequences were identified in *P. indica* genome. Three of these putative sugar transporters with accession number CCA70422.1, CCA68995.1, and CCA67897.1 were showing highest homology with query proteins. The predicted protein size of these putative 19 proteins was 568–487 amino acids. The putative function for two of these proteins with accession number CCA69504.1 and CCA70422.1 was assigned as 4-hydroxy benzoate transporter and quinate transporter, respectively in pedant database. For one other putative hexose transporter with accession number CCA68994.1, only a partial sequence of 67 amino acids is given in database and was found to be a hypothetical protein. Based on these data three sequences were rejected. These 16 putative sugar transporter sequences were further analyzed by SACS HMMTOP program for their membrane topology, we found that nine out of 16 protein sequences were showing typical MFS transporter membrane arrangement with 12 transmembrane helices, with a long intracellular loop between helix 6 and 7 and cytosolic N and C-terminal. Finally nine sequences were aligned using Multialin and ClustalW2 with hGLUT1, hGLUT2, hGLUT3, and XylE to find conserved regions in *P. indica* hexose transporter family. The hGLUT1 and XylE crystal structures were available that explained its functional motifs. Multiple sequence alignment shows presence of many conserved residues in these proteins. They all contain two conserved GR-[KR] motifs, which are present in MFS transporters and the PETKG sequence, which is highly conserved among sugar transporters. *P. indica* putative sugar transporters show 17–60% homology with other sugar transporters. Invariant and highly conserved sugar porter family signature motifs [GR]-[P]-[PESPR]-[V-GR]-[LFP]- [PETKG] are found in PiHXT5, PiHXT8, and PiHXT9 similar to hGLUT1-3 and XylE (**Figure 1**). The structural and functional significance of these motifs has been examined previously. The PiHXT5, PiHXT8, and PiHXT9 were found to exhibit high sequence similarity to hGLUT1-3 (**Figure 1**). This strongly suggests that these transporters are the member of the sugar porter family.

### *PiHXT5*, a Fungal Carbohydrate Transporter Expression is Induced during Colonization

**FIGURE 2:**
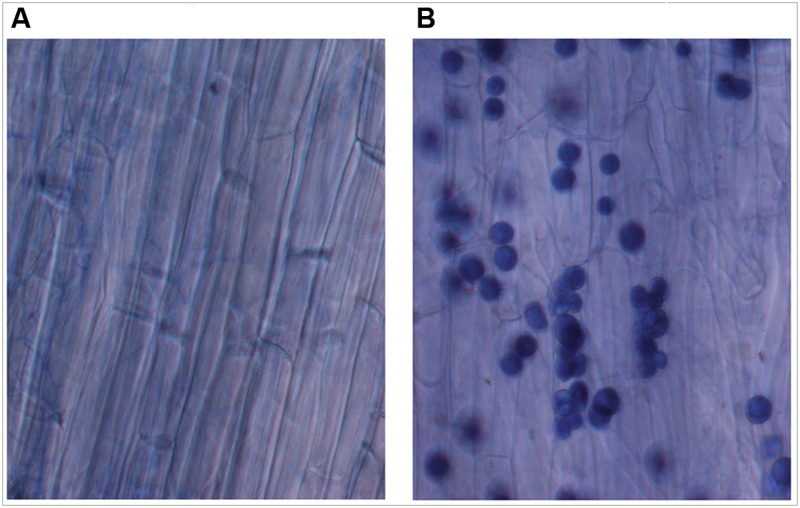
**Trypan blue staining of maize plant roots to show the colonization of maize roots by *P. indica*. (A)** Control maize roots without *P. indica*. **(B)** Maize root cortical cells showing intracellular chlamydospores of *P. indica* after 15 days of colonization.

**FIGURE 3:**
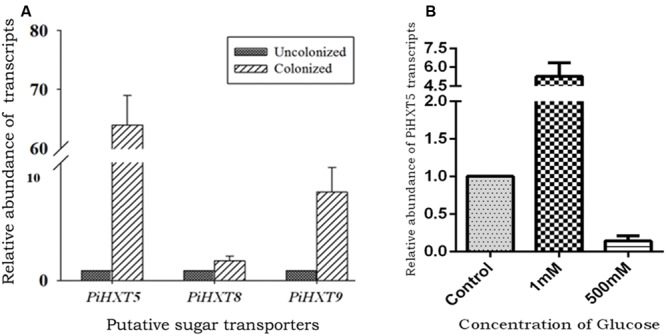
**Expression analysis of *PiHXT5* by qRT-PCR. (A)** Effect of colonization of *P. indica* with maize plant on *PiHXT5, PiHXT8, and PiHXT9* expression. qRT-PCR was performed to quantify relative expression level of *PiHXT5* in axenically grown *P. indica* and in colonized state of *P. indica* after 15 days of colonization at low phosphate (10 μM) in MN media. **(B)** Effect of different D-glucose concentrations on expression of *PiHXT5*. *P. indica* was grown in MN with different D-glucose concentrations (1 and 500 mM) for 5 days and qRT-PCR was performed to quantify *PiHXT5* relative expression level in different glucose concentrations, the expression was compared with control where *P. indica* was grown in 111 mM glucose. qRT-PCR was performed using gene specific primer and SYBR green I. The comparative C_t_ method was applied to analyze the data. For experimental samples, targeted quantity was determined and divided by the target quantity of the calibrator (*PiTef*). Thus, the calibrator becomes the 1X sample, and all other quantities are expressed as an *n*-fold difference relative to the *PiTef*.

Recently, plant–fungal symbiotic stage is reflected by induced expression pattern of specific AMF carbohydrate transporter as well as plant mycorrhiza specific phosphate transporter. It was shown during symbiosis, *Glomus* sp. monosaccharide transporter MST2 as well as plant phosphate transporter PT4 of *Solanum tuberosum* (St PT4) and *Medicago truncatula* (Mt PT4) gets induced as the marker of symbiotic stage. Interestingly, *Glomus* sp. monosaccharide transporter MST2, whose expression is undetected at spore stage, follows the expression pattern of PT4 during colonization. Similarly we inquired that, which sugar transporter of *P. indica* is induced during the colonizing stage, that may help the fungus to acquire carbohydrate from host plant. It was observed that *P. indica* colonization in plant root is a time-dependent process. Further we found 65% colonization at day 15 which was characterized by the presence of intracellular pear-shaped chlamydospores (**Figure 2**). To identify the hexose transporters that are regulated in response to mutualistic interaction with maize plant, qRT-PCR analysis was done. Out of nine sugar transporters, three (*PiHXT5, PiHXT8*, and *PiHXT9*) found to be upregulated during the colonization stage as compared to non-colonizing or axenically grown fungus. Further, as compared to *PiHXT8* and *PiHXT9, PiHXT5* expression was found to be highly elevated during colonization with host plant (**Figure 3A**). *PiHXT5* was found 64-fold up-regulated after 15 days of colonization as compared to non-colonized *P. indica*. However as compared to *PiHXT5*, both *PiHXT8* and *PiHXT9* expression were found to be non-significant (**Figure 3A**). Therefore, only *PiHXT5* was selected for further study.

### Glucose Deficiency Triggers the Expression of *PiHXT5*

We also investigated the effect of glucose on the expression of the *PiHXT5*. The normal glucose concentration in routine culture was established as 111 mM (or 2% w/v). Therefore, we compared the expression of *PiHXT5* at different glucose concentration, i.e., 1 and 500 mM with respect to 111 mM. Interestingly, very low glucose concentration (1 mM) was found to induce (five-fold) the expression of PiHXT5 in axenic culture as compared to optimum glucose level (111 mM). However, *PiHXT5* was found to be suppressed (10-fold) at higher glucose concentration (500 mM) as compared to optimum glucose level (111 mM). This expression pattern of *PiHXT5* suggests its high-affinity nature (**Figure 3B**).

### Isolation of CDS, 5′ and 3′ UTR

**FIGURE 4:**
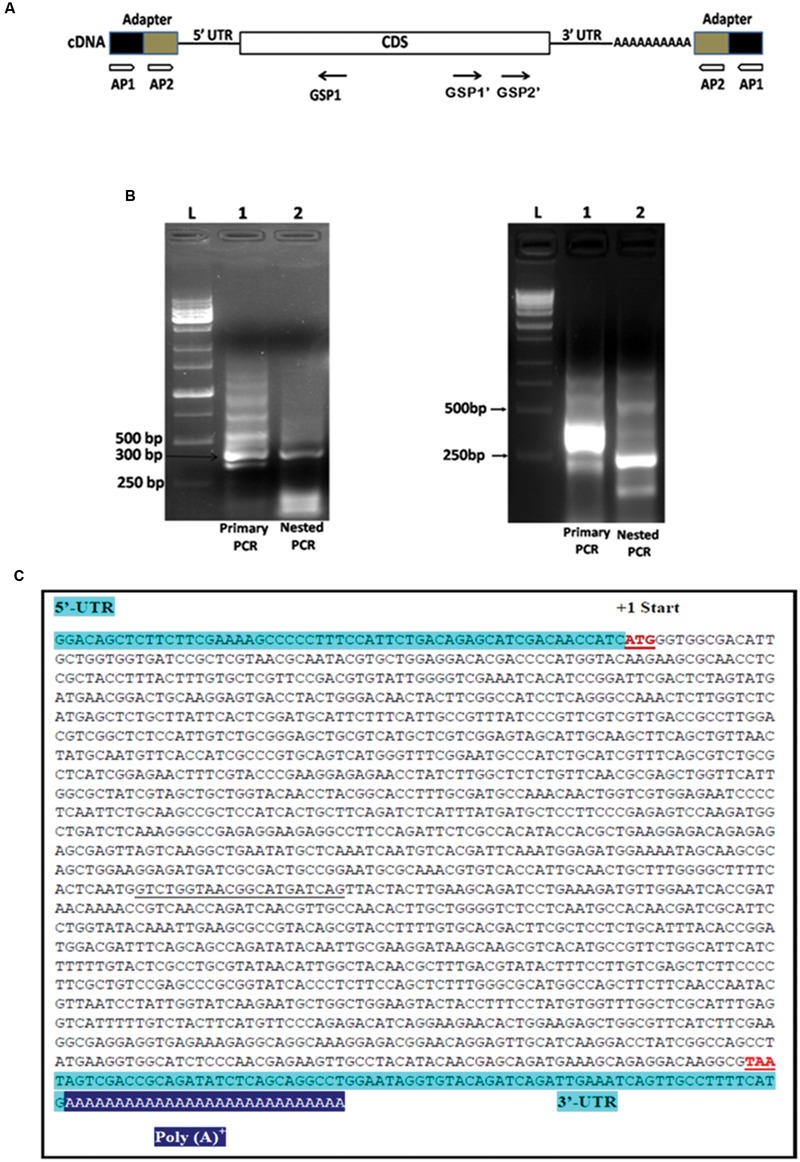
**Rapid amplification of cDNA ends (RACE) analysis for the full length gene of *PiHXT5*. (A)** Schematic presentation of 5′ and 3′ RACE amplification. Two RACE assays were conducted by using two adapter primers for both of 5′ RACE and 3′ RACE (AP1 and AP2) and *PiHXT5* GSP1 (nucleotides 312–331 bp) for 5′ RACE and GSP1′ (nucleotides 1288–1308 bp) and GSP2′ (nucleotides 1463–1483 bp) for 3′ RACE. In the first 5′ RACE primary PCR were set with AP1-GSP1 primer pairs (each of lanes 1 in figure **B**) and nested PCR with AP2-GSP1 (each of lanes 2 in figure **B**). In 3′ RACE the primary PCR and nested PCR were conducted with different set of primers AP1-GSP1′ and AP2-GSP2′, respectively. **(B)** Analysis of the primary and nested PCR products on agarose gel, The 1 Kb DNA size marker are shown in extreme left lane marked by letter L. **(C)** Full length sequence of *PiHXT5* gene of *P. indica.* Sequences of the *PiHXT5* 5′ UTR and 3′ UTR are shown in shaded letters. The first start codon (methionine) is numbered +1. The sequencing of cloned *PiHXT5* CDS shows the presence of 21 extra nucleotide (underlined) as compared to available CDS sequence of predicted *hexose transporter* (GenBank CCA71201.1).

The *PiHXT5* CDS was amplified, cloned and sequenced from cDNA of *P. indica*. The gene was found to be 1.599 Kb in length (**Figure 4**). The sequencing of cloned *PiHXT5* CDS shows the presence of 21 extra nucleotide (888–908 downstream of ATG) as compared to derived CDS sequence of predicted hexose transporter (GenBank CAFZ01000110.1) from *P. indica* genome sequence. *PiHXT5* encodes a polypeptide of 532 as compared to initially predicted 525 amino acids having a relative molecular mass of 59.69 kDa.

To isolate 5′ UTR, 5′ RACE analysis was performed by using reverse *PiHXT5* gene specific primers GSP1 (**Figure 4B**). Primary PCR was performed with outer primer AP1 and reverse primer GSP1 by using total RNA from *P. indica* which shows a multiple bands on the agarose gel (**Figure 4B**). The primary PCR products were used as template for nested PCR by using AP2 and GSP1. Single prominent product was obtained from nested PCR (**Figure 4B**). It can be predicted from this result that single transcriptional start site is present for *PiHXT5* gene. The 5′ RACE product was cloned into T-A cloning vector pGEM-T Easy. The sequencing of 10 different randomly selected clones shows that they have the same sequence. The first base of this product was therefore assigned position -57, and it is located 57 bp upstream of *PiHXT5* start codon (**Figure 4C**). Similarly, 3′ RACE analysis was performed by successive primary and nested PCR using AP1-GSP1′ and AP2-GSP2′ primers, respectively. The 3′ UTR product is 73 bp long excluding the poly A-tail downstream of *PiHXT*5 stop codon.

### Homology and Phylogenetic Analysis

**FIGURE 5:**
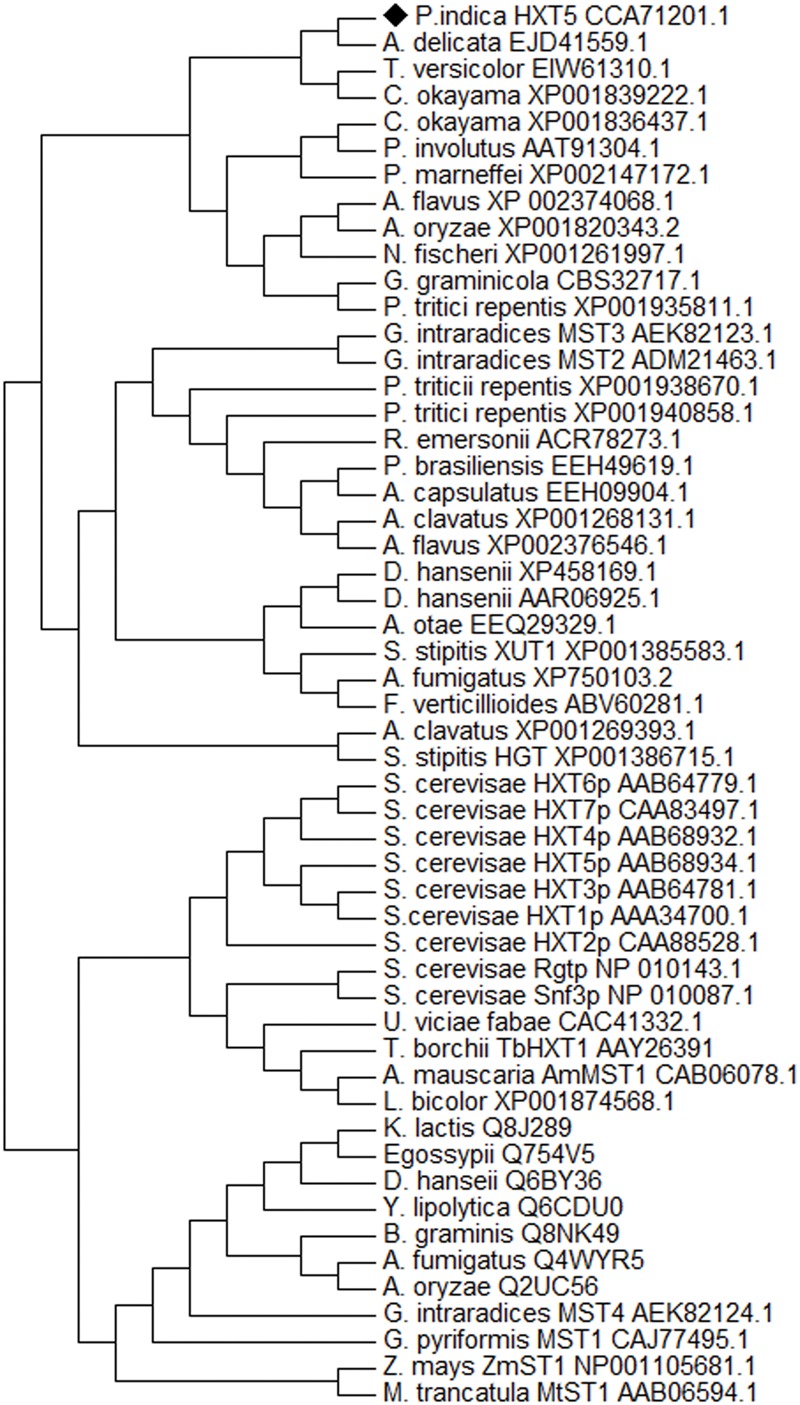
**Phylogeny of deduced protein sequence of PiHXT5.** Phylogenetic tree of fungal monosaccharide transporter protein sequences. The dendrogram was generated by Mega 5 software using the neighbor-joining method for the construction of the phylogeny (Saitou and Nei, 1987). Bootstrap test were performed using 1000 replicates. The branch lengths are proportional to the phylogenetic distance. The sequence of the sugar transporters ST1 from *Zea mays* and *Medicago truncatula* were used as outgroups. *P. indica* PiHXT5 cluster together with uncharacterized fungal hexose transporters from basidiomycota fungi.

**FIGURE 6:**
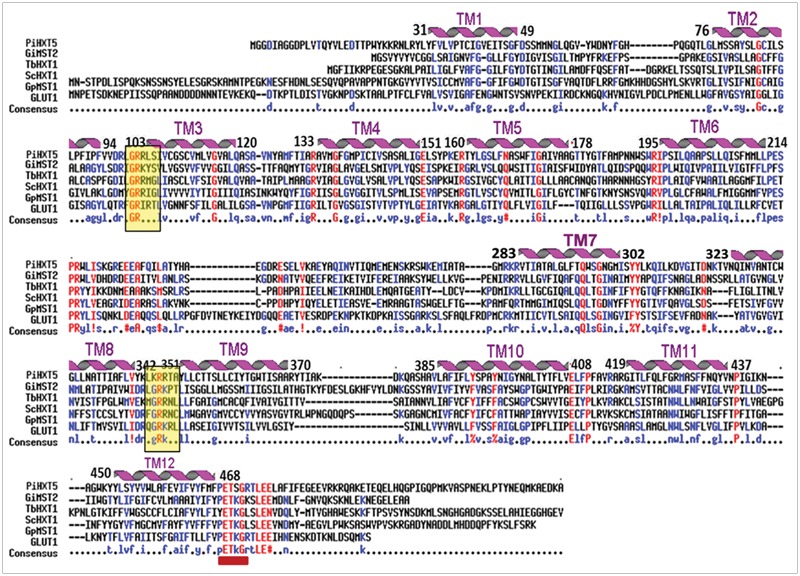
**Alignment of the predicted amino acid sequence of *PiHXT5* with *Glomus intraradices (GiMST2), Tuber borchii (TbHXT1), Saccaharomyces cerevisiae (ScHXT1)* and *Geosiphon pyriformis (GpMST1)* and *Homo sapiens (hGLUT1)* by using MULTIALIN.** The degree of sequence conservation at each position amino acids is shown in red, and low consensus amino acids are shown in blue. Membrane-spanning domains (TM) of PiHXT5 as predicted by HMMTOP are shown as helices over the corresponding amino acid sequences indicated by numerals (TM1–TM12). Prediction of functional motifs in PiHXT5 polypeptide was performed with PROSITE data base (available on-line). The PETKG sequence, which is highly conserved among sugar transporters, is indicated with red bar. Two GR-[KR] motifs typical of the transporters belonging to the MFS superfamily are highlighted by yellow color (Consensus symbols: “!” is any sequence of IV, “$” is any sequence of LM, “%” is any sequence of FY, and “#” is any sequence of NDQEBZ.).

Blastx analysis showed 57–75% identity with many putative fungal hexose transporters. It has 23% identity with other hexose transporters such as *GiMST2* (ADM21463.1), *GpMST1* (CAJ77495.1), *TbHXT1* (AAY26391), and *ScHXT1* (AAA34700.1; **Table 2**). Phylogenetic analysis suggests that *PiHXT5* cluster together with uncharacterized fungal hexose transporters from *Auricularia delicate* (EJD41559.1), *Trametes versicolor* (EIW61310.1), *Coprinopsis cinerea okayama* (XP001839222.1), *Paxillus involutus* (AAT91304.1), and *Aspergillus flavus* (XP002374068.1). These branches include closely related hexose transporters from basiodiomycota or ascomycota fungi (**Figure 5**). The putative membrane topology of *PiHXT5* was deduced by HMMTOP program which shows 12 transmembrane helices with cytosolic N and C-terminal, and a long intracellular loop between helix 6 and 7. PROSITE shows that *PiHXT5* belongs to MFS, we have identified two G-R-[KR] motifs identified in *PiHXT5* polypeptide, which are typical of these transporter proteins. The first one is located between 2 and 3 transmembrane domain and the second between transmembrane domains 8 and 9. Moreover, sugar transporter PFAM motif (PF0083) was also present in *PiHXT5* polypeptide at position 45–480 and MFS superfamily domain (PS50850) is present at position 36–471 (**Figure 6, Table 3**).

**Table 2:** Percentage homology between *PiHXT5* and hexose transporters from fungi, bacteria, plant, and animal.

Name of organism	Description	GenBank accession number	Homology with *PiHXT5* (%)	*E*-value	Query coverage %
*Piriformospora indica* (fungus)	*PiHXT5*	CCA71201.1	99	0.0	98
*Auricularia delicate* (fungus)	*Putative hexose transporter*	EJD41559.1	75	0.0	93
*Rhizoctonia solani 123E* (fungus)	*MFS sugar transporter*	KEP49025.1	68	0.0	95
*Gloepphyllum trabeum* (fungus)	*General substrate transporter*	XP007862033.1	63	0.0	92
*Trametes versicolor* (mushroom)	*Putative hexose transporter*	EIW61310.1	63	0.0	94
*Coprinopsis cinerea okayama* (mushroom)	*Putative hexose transporter*	XP001839222.1	63	0.0	90
*Dichomitus squalens* (fungus)	*Putative hexose transporter*	EJF62162.1	61	0.0	93
*Coniophora puteana* (fungus)	*Putative hexose transporter*	EIW76243.1	62	0.0	92
*Colletotrichum orbiculare* (fungus)	*Putative sugar transporter*	ENH85362.1	57	0.0	94
*Glomus intraradices* (AMF)	*GiMST2*	ADM21463.1	24	3e-46	94
*Geosiphon pyriformis* (AMF)	*GpMST1*	CAJ77495.1	23	7e-22	75
*Tuber borchii (fungus)*	*TbHXT1*	AAY26391	23	2e-21	84
*Saccharomyces cerevisiae* (yeast)	*ScHXT1*	AAA34700.1	23	3e-28	78
*Homo sapiens* (human)	*GLUT1*	GI 636666609	24	1e-17	92
*Zea mays* (Maize plant)	*ZmMST1*	NP001105681.1	24	6e-26	81

**Table 3:** Position of conserved domains in *PiHXT5* and other fungal hexose transporters.

Name of organism	Hexose transporter (number of amino acids)	Sugar transporter PFAM motif (PF0083)	MFS superfamily domain (PS50850)
*P. indica*	*PiHXT5*(532)	45–480	36–472
*G. intraradices*	*GiMST2*(494)	18–475	13–464
*G. pyriformis*	*GpMST1*(540)	63–527	63–516
*T. borchii*	*TbHXT1*(520)	23–472	21–462
*S. cerevisiae*	*ScHXT1*(569)	68–527	67–516
*H. sapiens*	*GLUT1*(504)	19–463	15–456

### Complementation Assay

In order to functionally characterize *PiHXT5* and to investigate its kinetic properties and substrate specificity, it was cloned in yeast expression vector p112A1NE under the control of ADH1 promoter. This construct p112A1NE-*PiHXT5* was transformed into *S. cerevisiae* strain EBY.VW4000. Because EBY.VW4000 is devoid of any hexose transporter, its growth was found retarded on glucose, as compare to EBY.VW4000 complemented with *PiHXT5* or its parental strain harboring only p112A1NE plasmid vector. Growth of EBY.VW4000 transformed with p112A1NE-*PiHXT5* construct or empty expression vector and untransformed EBY.VW4000 was compared in YNB medium containing all the amino acids except tryptophan and 2% D-glucose as carbon source at OD_650_ for 2 h at 30°C. The expression of *PiHXT5* cDNA in mutant yeast enabled the mutant to grow significantly as compare to mutant harboring only p112A1NE vector and untransformed EBY.VW4000.

### PiHXT5 is a Broad Substrate Range Monosaccharide Transporter

**FIGURE 7:**
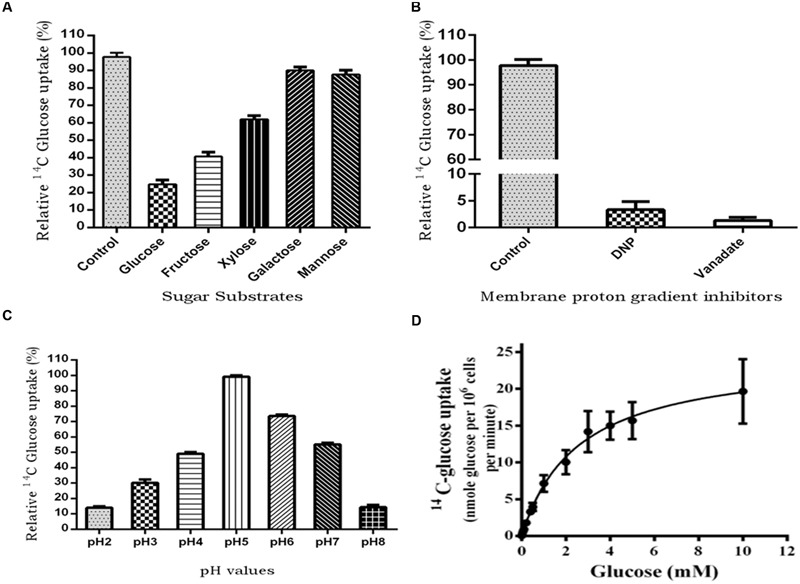
**^14^C-glucose uptake in *PiHXT5*-expressing yeast strain EBY.VW4000. (A)** Substrate competition on the respective sugars; *PiHXT5* is able to transport D-glucose (highly), D-fructose, and D-xylose (moderate). It was weakly able to transport D-galactose and D-mannose. **(B)** Incubation of *PiHXT5* expressing yeast cells in presence of protonophore (DNP) and plasma membrane H^+^-ATPase inhibitor (vanadate) for 5 min strongly inhibits glucose uptake. **(C)** The optimum pH for glucose uptake by *PiHXT5* is pH5 and highest uptake is found between pH 5 and 6. The readings are relative to negative control (^14^C-glucose transport in strain EBY.VW4000 transformed with vector only; **D**) Michaelis–Menten kinetics of glucose uptake rates (pH 6.5) indicates a K_m_ of 2.56 ± 0.18 mM and V_max_ of 24.5 ± 0.69 nmol/min/10^6^ cells. Error bars represent SD; *n* = 3.

To characterize PiHXT5 biochemically, we expressed PiHXT5 in glucose transport deficient mutant of *S. cerevisiae* EBY.WV4000. Glucose uptake by EBY.VW4000 harboring *PiHXT5* was further confirmed by measuring uptake of ^14^C-glucose. EBY.VW4000 expressing *PiHXT5* accumulated ^14^C-glucose at a rate significantly higher than the background rate shown by the control EBY.VW4000 cells. To investigate the substrate specificity, competition experiment was performed, where glucose uptake was challenged with five-fold molar excess of competitive sugar. The competition assay was performed in presence of D-glucose, D-fructose, D-mannose, D-galactose, and D-xylose. Using ^14^C-labeled sugars, we showed that PiHXT5 is able to transport Glc, Fru, Xyl, Man, and Gal with decreasing affinity in that order (**Figure 7A**).

The ^14^C-glucose uptake rate of PiHXT5 was found significantly lower in presence of protonophore DNP and plasma membrane H^+^-ATPase inhibitor sodium vanadate as compared to control (absence of these chemicals; **Figure 7B**). The PiHXT5 lose its transport property in the presence of protonophore, which indicates its secondary nature of H^+^ co-transport. To obtain the optimum pH value for function of PiHXT5, EBY.VW4000 expressing *PiHXT5* was subjected to ^14^C-glucose transport at different pH value ranging from pH 2 to 8. We found that glucose transport activity of *PiHXT5* is pH dependent. The optimum value for glucose transport by *PiHXT5* is between pH 5 and 6 (**Figure 7C**). Affinity studies show that the apparent K_m_ of PiHXT5 is 2.56 ± 0.18 mM. The K_m_ is closest to the highest affinity monosachharide transporter reported so far. The glucose uptakes by cells expressing *PiHXT5* follow Michaelis–Menten kinetics with an apparent K_m_ of 2.56 ± 0.18 mM (V_max_ 24.5 ± 0.69 nmol/min/10^6^ cells), which suggests it is a high-affinity transporter (**Figure 7D**).

## Discussion

The carbon flow from the photoautotrophic plants to fungal heterotrophs plays the key role in the formation of symbiotic relationship (Ho and Trappe, 1973). The mechanism of transportation of photosynthate from the host plant cortical cells to the fungal tissue involves three major steps (1) Release of sucrose into apoplastic space by gradient diffusion (2) Degradation of sucrose by cell wall invertase into simpler molecules like fructose and glucose (3) Subsequent, transport across fungal membrane into the intra-radical hyphae (Gianinazzi-Pearson, 1996; Hodge et al., 2010). The mechanism of carbohydrate transported across symbiotic interface is still unexplored. Monosaccharide transporters plays critical role in transportation of nutrients across plasma membrane (Lalonde et al., 2004). It would be of utmost importance to identify and characterize the fungal transporters involved in uptake of carbohydrate from the symbiotic interface for the survival and maintenance of the fungal partner. In this study, we have analyzed putative hexose transporters from genome of mutualistic endophyte *P. indica*, that confers nutrient enrichment (e.g., N, P, K, S, Mg, Co, Cu, Zn, etc.) in plant under deprived conditions (Varma et al., 1999; Yadav et al., 2010). There are 19 putative hexose transporters in *P. indica* genome, which shows 15–91% homology with each other. Similar number of hexose transporters were reported in *Laccaria bicolor* (López et al., 2008), *S. cerevisiae* (Ozcan et al., 1998; Fan et al., 2002), *Candida albicans* (Fan et al., 2002). The presence of GR-[KR] motifs conserved in MFS transporters and the PETKG sequence, signature tag of sugar transporters (Pao et al., 1998; Sun et al., 2012; Deng et al., 2014) in PiHXT5 assign it to sugar porter family.

We reported the role of AMF like fungus *P. indica* hexose transporter during symbiosis, having the ability to transport glucose as well as plant cell wall derived sugars. Till date, only report available related to the hexose transporters from AMF which are involved in the plant symbiosis is *MST2* from *G. intraradices* (Helber et al., 2011).

Our study reveals the role of symbiotic signaling in the regulation of fungal Hexose transporter and subsequent carbohydrate supply to *P. indica* when it colonizes the host plant. Using the benefit of axenic culture of *P. indica* on synthetic media, we are able to differentiate the expression pattern of fungal carbohydrate transporters in presence and absence of symbiotic signals (Harrison, 1999, 2005). Thus, it deciphers the effect of mutualism on the expression pattern of hexose transporters. In our study *PiHXT5* is 64-fold upregulated during colonization as compared to axenic state. Our data supports the previous study, in which MST2 from *G. intraradices* have shown to be induced during symbiosis (Helber et al., 2011). Further, we have a novel finding that the hexose transporters are regulated by extracellular glucose concentration. Low glucose concentration induced the expression of *PiHXT5*. This may be explained by the fact that rate of photosynthesis is regulated by many environmental factors which affects the carbohydrate supply to the sink tissues like root cortical cells. Subsequently, there may be decrease in concentration of sugar in apoplast (Sonnewald et al., 1997), therefore to maintain the constant C flux, fungus adapts by inducing hexose transporter expression. Similar reports were present on glucose transporters in *S. cerevisiae* (Carlson, 1999), but not in mutualistic fungus. Present work will open some novel questions like how the mycorrhizal glucose transfer mechanism works in apoplast? Is photosynthetic activity of the plant determines the transcriptome of the symbiotic fungus? Is symbiotic signaling and glucose signaling is co-related? These questions needs warrant investigation.

Further, the PiHXT5 also shows a broad range of substrate specificity. The result is in agreement with previous study which showed the versatility of MST2 as a sugar transporter. It transports multiple substrates such as xylose, glucuronic, galacturonic acid, mannose, and galactose efficiently, beside glucose (Helber et al., 2011). Similarly, the sugar transporter from *G. pyriformis* have shown that mannose was more efficiently transported than glucose (Schubler et al., 2006). This result suggests like MST2, PiHXT5 might transport not only glucose but also cell wall constituting monosaccharides. Previous cytological and biochemical studies have shown the presence of non-assembled primary plant cell wall components, such as β-1,4-glucans, xyloglucans, non-sterified polygalacturonans, and arabinogalactan proteins at the plant–fungal interface in AM symbiosis (Perotto et al., 1995; Gianinazzi-Pearson, 1996). This implies that during C starvation, the fungus can feed on “amorphous cell wall components” mainly glucose, mannose, galactose, xylose, and arabinose (York et al., 1986). It has been reported that the AMF is able to feed on cell wall components (Ho and Trappe, 1973; Hodge et al., 2010). Other studies have shown systemic activation of a plant xyloglucan endotransglucosylase/hydrolyase gene (XTH) in mycorrhizal roots (Maldonado-Mendoza et al., 2005), xyloglucanase capacity of AMF (Rejon-Palomares et al., 1996) which supports the above theory. Similar observations were reported from *GpMST1* from cyanobacterial symbiotic fungus *G. pyriformis* and *MST2* from *G. intraradices* (Redecker and Raab, 2006; Helber et al., 2011), both of these proteins are high affinity glucose transporters with a broad substrate specificity for different monosaccharides. This adaptation of acquiring C from cell wall components suits well for biotrophic modus of life.

PiHXT5 protein is expressed in heterologous system leading to functional complementation. Since we did not get the spot assay, phenotypic complementation was not observed, similar results were obtained by Helber et al. (2011). We found that PiHXT5 is a high- affinity glucose transporter as it has K_m_ value 2.5 mM and V_max_ of 24 nmol/min/10^6^ cells. Other high-affinity glucose transporters of fungus and plants found to have similar K_m_ and V_max_, e.g., fungal *GpMST1* has K_m_ of ∼1.2 mM and *Uromyces fabae HXT1* has K_m_ 0.36 mM (Voegele et al., 2001) and TbHXT1 has K_m_ 38 μM and *Glomus MST2* has K_m_ 33 μM for glucose thus support our findings. Most highly expressed sugar transporter like STP1, STP4, STP7, and STP13 from *Arabidopsis thaliana* (Büttner, 2010) reported to have K_m_ 15–74 μM. The high affinity nature of PiHXT5 is consistence with data from qRT-PCR as its expression was higher at low glucose levels. Similar reports are presents showing how eukaryotic cells sense availability of glucose, and through glucose sensors (Snf3 and Rgt2) generate a signal for altering the expression of hexose transporters in *S. cerevisiae* (Ozcan and Johnston, 1999). Radioactive sugar uptake experiments using ^14^C-glucose shows that yeast cells expressing *PiHXT5* were able to transport glucose. Substrate specificity experiments using ^14^C-glucose with five-fold molar excess of D-fructose, D-xylose, D-galactose, and D-mannose shows that *PiHXT5* has highest specificity for D-glucose, although it can also transport D-fructose and D-xylose, which are components of plant cell wall. pH dependence studies of *PiHXT5* shows that the maximum transport activity of radioactive glucose was found between pH 5 and 6. The glucose transport activity of *PiHXT5* dependent on membrane proton gradient and also on H^+^-ATPases as glucose uptake was found blocked in presence of protonophore DNP and plasma membrane H^+^-ATPase inhibitor sodium vanadate, similar results were also found in case of *MST2* from *G. intraradices* and *GpMST1* from *G. pyriformis*, HXT1 from *U. fabae* and also in case of three hexose transporters *HXT B, C*, and *E* from *A. nidulans*, where glucose transport was found sensitive to protonophore CCCP (Carbonyl Cyanide 3-ChloroPhenylhydrazone), suggesting that they function as energy dependent H^+^/glucose symporters (Requena et al., 2003). We suggest that PiHXT5 uptake monosachharides using the H^+^ gradient generated by this enzyme. The present study provides functional and biochemical characterization of PiHXT5 from *P. indica*. Our study will open new vistas to understand the glucose transport and metabolism in this symbiotically important fungus. The characterization of first hexose transporter from this fungus will also help in understanding the nutrient exchange mechanism between plants and *P. indica*.

## Author Contributions

MR, SR, MK, and VD have performed the experiments. MK, SR, AJ, and MD have designed the experiments. Chemicals were provided by AJ and MD. Project was supervised by AJ and MD. MS is written by AJ and MD.

## Conflict of Interest Statement

The authors declare that the research was conducted in the absence of any commercial or financial relationships that could be construed as a potential conflict of interest.

## Footnotes

1 www.ncbi.nlm.nih.gov

2 http://www.ebi.ac.uk/Tools/msa/clustalw2

3 http://pedant.gsf.de/
